# The transjugular approach is a safe and effective alternative for performing portal vein embolization

**DOI:** 10.1097/MD.0000000000017851

**Published:** 2019-11-11

**Authors:** Ming-Shan Jiang, Xue-Feng Luo, Zhu wang, Xiao Li

**Affiliations:** a Department of Gastroenterology; b Institution of Interventional Radiology, West China Hospital, Sichuan University, Chengdu, Sichuan, China.

**Keywords:** hepatocellular carcinomas, internal jugular vein, interventional oncology, portal vein embolization, transjugular intrahepatic portosystemic shunts

## Abstract

To evaluate the safety and efficacy of the novel technique, transjugular portal vein embolization (TPVE).

A single-center retrospective review of 18 patients (12 males and 6 females; mean age, 62 years) who underwent TPVE between January 2012 and January 2013 was conducted. The technical success rate, future liver remnant (FLR) volume, total liver volume (TLV) and FLR/TLV ratio after PVE were analyzed. Liver function, including total bilirubin (TB), aspartate aminotransferase (AST), alanine aminotransferase (ALT) and International Normalized Ratio (INR), was assessed before and after PVE. Any complications of TPVE and liver resection after TPVE were recorded.

TPVE was performed on 18 patients before right hepatic resection for both primary and secondary hepatic malignancies (10 hepatocellular carcinomas, 4 cases of colorectal liver metastasis, and 4 cholangiocarcinomas). Technical success was achieved in 100% of patients (18 of 18). The mean FRL significantly increased to 580 ± 155 mL (*P* < .001) after PVE. The mean FLR/TLV ratio (%) significantly increased to 34 ± 4 (*P* < .001) after PVE. One patient suffered septicemia after TPVE. A small number patients experienced mild to moderate abdominal pain during TPVE. No other major complications occurred after TPVE in our study. The patient who developed septicemia died 3 days after the surgery as a result of this complication and subsequent multiple organ dysfunction syndrome (MODS).

Transjugular portal vein embolization is a safe, efficacious, and promising novel technique to induce hypertrophy of the FLR.

## 1 Introduction

Hepatectomy is considered to support the long-term survival of patients with primary or secondary hepatic malignancies, such as hepatocellular carcinoma (HCC), cholangiocarcinoma or hepatic metastases.^[1–5]^ However, the postoperative hepatic failure is still the major cause of death following major liver resection and the main reason for hepatic failure is insufficient remaining liver volume. Hepatectomy can be considered safe when the future liver remnant (FLR) volume is >20.0% in patients with healthy livers and >31% to 40% in patients with impaired liver function, steatosis, or a history of hepatotoxic chemotherapy treatment. An FLR of at least 40% is recommended in patients with cirrhotic livers disease.^[6]^ To overcome this issue, portal vein embolization (PVE) has been used to induce hypertrophy of FLR before major hepatectomy as occlusion of one branch of the portal vein could results in hemodynamic changes and the upregulation of various humoral mediators, leading to the hypertrophy of contralateral segments and atrophy of ipsilateral segments.^[7,8]^

PVE consists of occluding the portal branches of the segments that will be resected; the portal flow is then abruptly and entirely redistributed toward the FRL's portal branches.^[9]^ Several techniques have been reported, including intraoperative portal branch ligation,^[10,11]^ transileocolic PVE,^[12]^ trans-splenic PVE^[13]^ and percutaneous ipsilateral or contralateral PVE.^[14,15]^ All of the above techniques have certain advantages and disadvantages, such as portal ligation require general anesthesia and obtain the risk of postoperative adhesions. Based on our high level of experience with transjugular intrahepatic portosystemic shunts (TIPS),^[16]^ we aimed to achieve PVE through the right internal jugular vein (TPVE) to investigate the methodology of this procedure.

## 2 Materials and methods

### 2.1 Patient characteristics

Between January 2012 and January 2013, a single-center retrospective review of our institutional database was performed with approval from our institutional review board. Patients with FLR <20% of the estimated total liver volume (TLV) or < 40% PVE were regarded as having high risk of liver failure after major hepatectomy and were referred to our unit for the PVE procedure. In total, 18 of the patients who underwent TPVE were included in this study. Four patients had cholangiocarcinoma and underwent percutaneous transhepaticcholangial drainage (PTCD) if additional time was considered necessary before TPVE. Five patients were treated with TACE (transcatheter arterial chemoembolization) one week before TPVE. All the patients involved had consented the study.

### 2.2 The PVE technique

All procedures were performed or supervised by two experienced interventional radiologists. After local anesthesia with lidocaine, catheterization of the hepatic vein was performed through the right internal jugular vein with a Rösch-Uchida transjugular liver access set (RUPS-100; Cook, Bloomington, IN). Direct portography was performed after the target intrahepatic portal branch was accessed. According to the planned surgery, the portal veins feeding the liver segment to be resected were embolized using coils (Cook Medical, Bloomington, IN) and/or polyvinyl acetate particles (300–500 um, Cook Medical, Bloomington, IN). The end point of embolization was blood stasis. Final portography was performed, and the catheter was pulled out. Patients were transferred to surgical ICU for three days.

**Figure 1:**
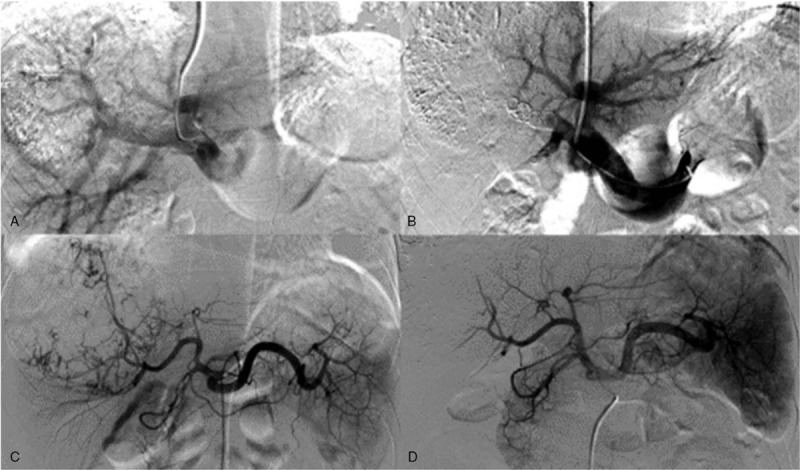
Digital subtraction angiogram image of a 68-year-old male with HCC. (A) Portography shows the hepatic artery before TACE. (B) Hepatic artery after TAVE. (C) Portography shows the portal vein. (D) Portography shows the occlusion of the right portal vein with continued patency of the veins supplying the left lateral liver (arrows).

Five patients were treated with transcatheter arterial chemoembolization (TACE) 1 week before TPVE. A 5F catheter was inserted into the common hepatic artery, and a super-select proper hepatic artery after the tumor vessel was determined through angiography. Then chemotherapeutic agents (5-fluorouracil, cisplatin, and epirubicin) were injected (Fig. 1). Four patients were treated PTCD when additional time was considered necessary before TPVE.

### 2.3 Liver volume

**Figure 2:**
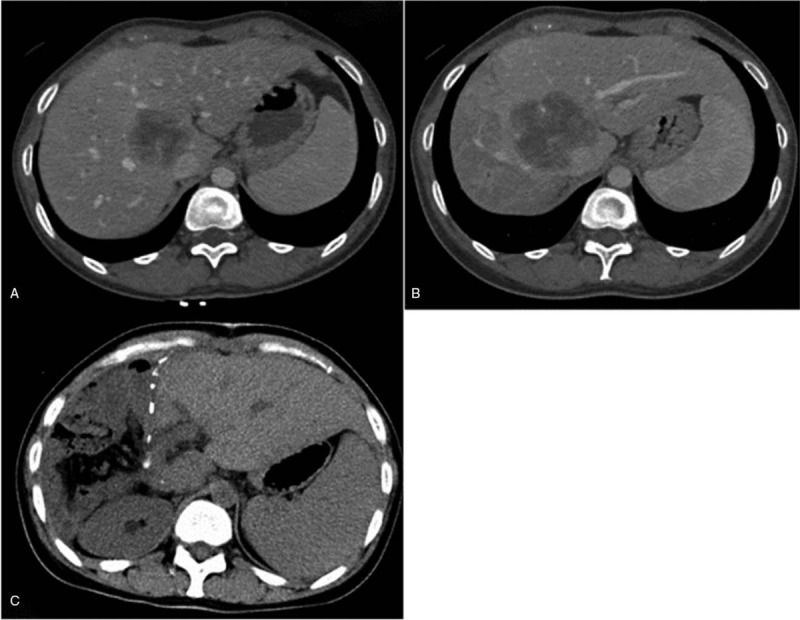
Contrast-enhanced CT image of the abdomen in a 61-year-old female with a hepatocellular carcinoma. (A) Focus on the right hepatic lobe (FLR/TELV, 36%). (B) Contrast-enhanced CT image obtained after TPVE, demonstrating hypertrophy of the FLR (FLR/TELV, 40%). (C) CT image obtained after the right hepatectomy.

All of the patients underwent a series of abdominal dynamic CT scans (Fig. 2) after the intravenous administration of contrast media at a mean of 10 days (range 7–18) before and 24 days (range 18–32) after PVE. The total liver volume (TLV) was calculated using the body surface area (BSA) with a previously described formula: −794 + 1267.283 × BSA.^[17]^

The %FLR was calculated as follows: 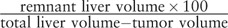 The increase in the percentage of remnant liver volume was calculated as follow: 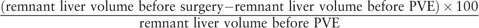 .^[18]^

### 2.4 Liver function

The biochemical parameters of liver function, including total bilirubin (TB), aspartate aminotransferase (AST), alanine aminotransferase (ALT) and International Normalized Ratio (INR) were documented before and after (2 to 21 days) the PVE procedure.

### 2.5 Statistical analysis

Paired Student's *t* tests were used to analyze the differences in pre- and post-embolization liver enzyme levels and the changes in liver volume. All analyses were performed using SPSS statistical software (version 21.0; SPSS, Chicago, III), with a *P* value <.05 indicating statistical significance.

## 3 Results

The subjects included 12 male and 6 female patients with a mean age of 62 (range: 50–75). Of the 18 patients treated with TPVE, 15 had healthy livers with tumor; 1 had steatosis; and 2 had Child-Pugh class A cirrhosis. The pathology of the underlying liver disease in the treated patients included hepatocellular carcinoma (n = 10), colorectal cancer liver metastases (n = 4) and cholangiocarcinoma (n = 4). All diagnoses were made by imaging findings and confirmed by cytology or histopathology. The demographics of the patients and liver volume data before and after PVE are provided in Table 1.

**Table 1:**
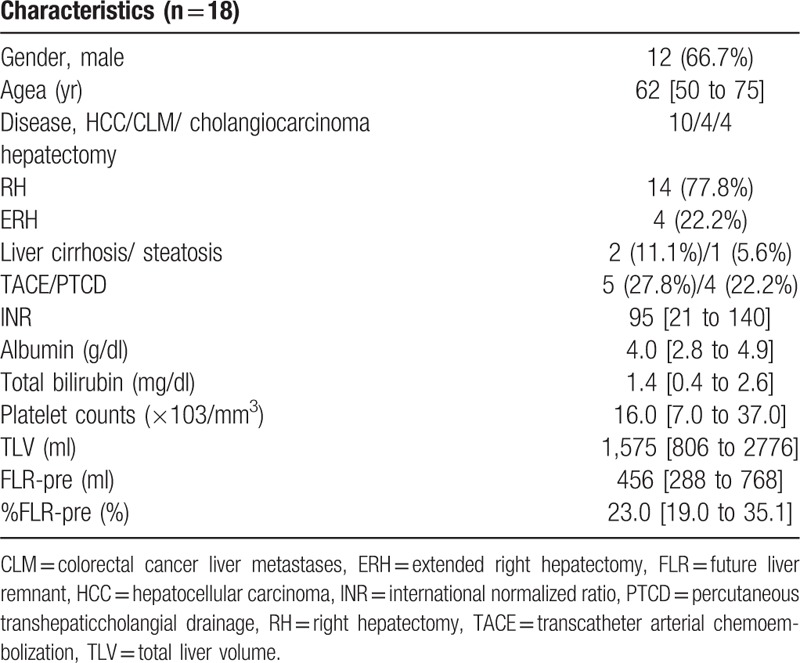
Clinical characteristics of the patients.

TPVE was successful in 18 of 18 (100%) patients. The right hepatic segment was embolized in each patient. The access site was located in right portal vein in 18 patients. Only one patient which had cholangiocarcinoma incurred major complications, developing high fever after TPVE; according to the results of a hemoculture, the patient was confirmed to have septicemia. After 3 weeks of antibiotic treatment, the patient's body temperature returned to baseline. One patient died three days after the surgery due to septicemia and subsequent MODS, even though the FLR increased from 479 ml to 590 ml after TPVE. Regarding minor procedural complications, a few patients experienced mild to moderate the abdominal pain during TPVE, which subsided before the end of TPVE without administration of additional analgesics. Two patients had a >1.0°C increase in body temperature after the TPVE had returned to a normal range, with or without antipyretics, within 3 days.

The evaluation of the postoperative function of the remaining liver parenchyma comprised evaluations of the total bilirubin (TB), aspartate aminotransferase (AST), alanine aminotransferase (ALT) levels and International Normalized Ratio (INR). Statistical analysis showed that there were no significant differences in the observed TB, AST, ALT or INR. All differences in the liver function parameters following PVE are provided in Table 2.

**Table 2:**
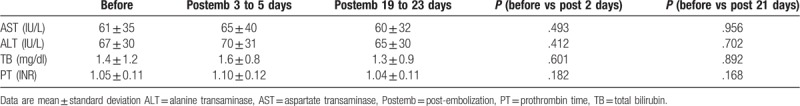
Differences in the liver function parameters following PVE.

Differences in the liver volume following embolization are proevided in Table 3. Mean FRL (ml) significantly increased from 456 ± 177 to 580 ± 155 after PVE (*P* < .001). Similarly, the mean FLR/TLV ratio (%) significantly increased from 23 ± 5 to 34 ± 4 after PVE (*P* < .001)

**Table 3:**
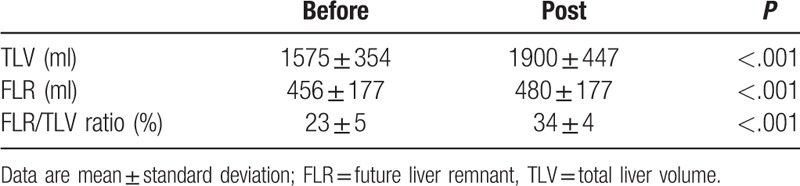
Differences in the liver volumes following embolization.

The median interval between PVE and surgical resection was 27 days (range, 20–65 days). All 18 patients achieved effective resections. Fourteen patients underwent a right hepatectomy, and four patients underwent a planned extended right hepatectomy.

## 4 Discussion

The first study on clinical PVE was published in 1986 by Kinoshita, who observed the atrophy of the hepatic lobes in which they embolized the portal branches to limit the intraportal extension of hepatocellular carcinoma.^[19]^ Since then, many articles have been published on this subject. Numerous studies have now shown PVE to be safe and efficacious for producing hypertrophy with a low risk of postoperative liver failure.^[7,20–23]^ Besides, PVE could also potentially increases the number of patients with initially unresectable HCC who can be offered resection^[21]^ and does not affect long-term survival in patients with HCC if the planned subsequent hepatectomy could be completed^[24]^ and does not affect the long-term survival and risk of cancer recurrence among colorectal liver metastases patients.^[25]^ Moreover, a study mentioned PVE could reduce postoperative hepatic insufficiency associated with postchemotherapy hepatic atrophy.^[26]^ In recent decades, TIPS has been widely used to treat the symptomatic complications of portal hypertension refractory to medical therapy. The rate of procedure related complications has decreased under experienced hands. Transjungular intrahepatic access to the portal vein system is considered a safe and useful approach.

PVE can be performed using trans-ileocolic, trans-splenic, ipsilateral, or contralateral approaches. With the increasing availability of radiological intervention suites, the percutaneous transhepatic technique has become the standard technique for PVE. The trans-ileocolic approach is a surgical procedure that is performed in the operating room under general anesthesia. However, this surgical procedure has generally been replaced by the less invasive percutaneous contralateral and ipsilateral techniques, which are accomplished using ultrasound-guided transhepatic punctures. The contralateral approach aims to puncture the portal system through the FLR. Because of the fewer acute angles between the access and target portal branches, this technique provides more favorable orientation for easier catheter manipulation toward the tumor-bearing liver. Furthermore, the segment 3 branches are commonly targeted because their anterior position allows for easier percutaneous access and less acute angles for right portal vein embolization. Still, the contralateral approach risks damaging the FLR due to iatrogenic trauma or nontarget embolization. The ipsilateral approach involves percutaneous access through the tumor-bearing liver, thereby avoiding potential damage to the FLR during instrumentation. This access allows for the easy catheterization of the segment 4 branches when they must be embolized. However, this approach involves access close to tumors in the ipsilateral lobe and requires care to avoid access through the malignant lesion, especially in large tumors. The acute angles made this technique difficulty of access to the right portal branches in a retrograde fashion. Additionally, the difficulty of finding a route through the healthy liver to the right portal branches is sometimes heightened. Therefore, both transhepatic approaches risk changing a patient's eligibility for potentially curative surgery and rendering the tumor inoperable.^[13]^ Manipulators must be careful to avoid accessing through the tumor to prevent peritoneal seeding.^[27–32]^

In our study, we decided to perform the PVE technique through the right internal jugular vein based on our rich experience with transjugular intrahepatic portosystemic shunts (TIPS), to determine the feasibility and potential advantages and disadvantages of this technique. Compared with the ipsilateral or contralateral approache, TPVE is much easier for interventional radiologist to manipulate catheter toward the tumor-bearing liver and easier access to the segment 4 branches. Moreover, as TPVE is performed through the right internal jugular vein, it has the advantage of no risk of damage to the FLR during access or catheter manipulation and reducing the risk of hemorrhage. Fortunately, the outcomes were quite encouraging as TPVE was successful in 18 of 18 (100%) patients. The mean percentage increase in the ratio of FLR to TLV after PVE was 11.0 ± 3.9%. Past studies of preoperative PVE with other techniques have reported mean percentage increases in the FLR/TELV ratio of 6% to 13%.^[18,33]^ Therefore, our results are consistent with those reported in previous studies. A few patients experienced mild to moderate abdominal pain during TPVE. No other major complications arose after TPVE in our study. A 50-year-old male (5.5%) with cholangiocarcinoma, experienced a high fever after TPVE; according to hemoculture, he was confirmed with septicemia had a white cell blood (WBC) increased to 12 × 10^9 /L. After 3 weeks of antibiotic treatment, his body temperature had returned to baseline. A 68-year old female with Child-Pugh class A cirrhosis died 3 days after the surgery due to septicemia and subsequent MODS. Her FLR increased from 479 to 590 ml 30 days after TPVE, and her liver function before surgery was quite normal. One day after her extended right hepatectomy, she experienced a high fever, and a blood smear indicated Gram-negative bacteria, and so we administered carbapenem antibiotics. Unfortunately, her TB, ALT, creatinine and brain natriuretic peptide levels increased to 43.9 mg/dl, 892IU/L, and 10.2 mg/dl, 14000pg/ml, respectively. Her blood gas analysis showed PH 7.15 and hyperpotassemia with serum potassium at 7.0 mmol/L. We administered hematodialysis. On the third day after surgery, she died due to MODS. We believe that septicemia caused multiple organ dysfunction. According to previous studies, complications due to PVE include subscapular hematoma, bile duct damage, hemoperitoneum, cholangitis, non-target embolization, recanalization of the segments that received embolization and complete portal vein thrombosis. The transcatheter embolization guidelines established by the Society of Interventional Radiologists suggested a threshold for PVE-related major complications of 6% and a threshold for PVE-related morbidity of 11%.^[34]^ According to Di Stefano's review, a total of 188 patients who underwent PVE via the contralateral approach produced 24 (12.8%) adverse events without mortality. Transient liver failure occurred at a significantly higher rate in patients with cirrhosis (5 of 30, *P* < .001).^[28]^ Our complication rates and mortality are well below this range.

The major drawbacks of our study include the lack of a control group, the small number of patients and the insufficient length of the follow-up period.

In conclusion, processing PVE through the internal jugular vein is a safe, efficacious, and promising novel technique to induce hypertrophy of the FLR.

## Author contributions

**Data curation:** Mingshan Jiang, Zhu Wang.

**Formal analysis:** Mingshan Jiang.

**Project administration:** Xue Feng Luo.

**Supervision:** Xue Feng Luo.

**Writing – original draft:** Mingshan Jiang.

**Writing – review & editing:** Mingshan Jiang.

Mingshan Jiang orcid: 0000-0001-5200-313X.
